# Characterization of C-nucleoside Antimicrobials from *Streptomyces albus* DSM 40763: Strepturidin is Pseudouridimycin

**DOI:** 10.1038/s41598-019-45375-w

**Published:** 2019-06-20

**Authors:** Petja Rosenqvist, Kaisa Palmu, Ranjit Kumar Prajapati, Keith Yamada, Jarmo Niemi, Georgiy A. Belogurov, Mikko Metsä-Ketelä, Pasi Virta

**Affiliations:** 1 Department of Chemistry, University of Turku, FIN-20014 Turku, Finland; 2 Department of Biochemistry, University of Turku, FIN-20014 Turku, Finland

**Keywords:** Chemical biology, Biochemistry

## Abstract

Pseudouridimycin (PUM), a selective inhibitor of bacterial RNA polymerase has been previously detected in microbial-extracts of two strains of *Streptomyces* species (strain ID38640 and ID38673). Here, we isolated PUM and its deoxygenated analogue desoxy-pseudouridimycin (dPUM) from *Streptomyces albus* DSM 40763, previously reported to produce the metabolite strepturidin (STU). The isolated compounds were characterized by HRMS and spectroscopic techniques and they selectively inhibited transcription by bacterial RNA polymerase as previously reported for PUM. In contrast, STU could not be detected in the cultures of *S. albus* DSM 40763. As the reported characteristics reported for STU are almost identical with that of PUM, the existence of STU was questioned. We further sequenced the genome of *S. albus* DSM 40763 and identified a gene cluster that contains orthologs of all PUM biosynthesis enzymes but lacks the enzymes that would conceivably allow biosynthesis of STU as an additional product.

## Introduction

**Figure 1:**
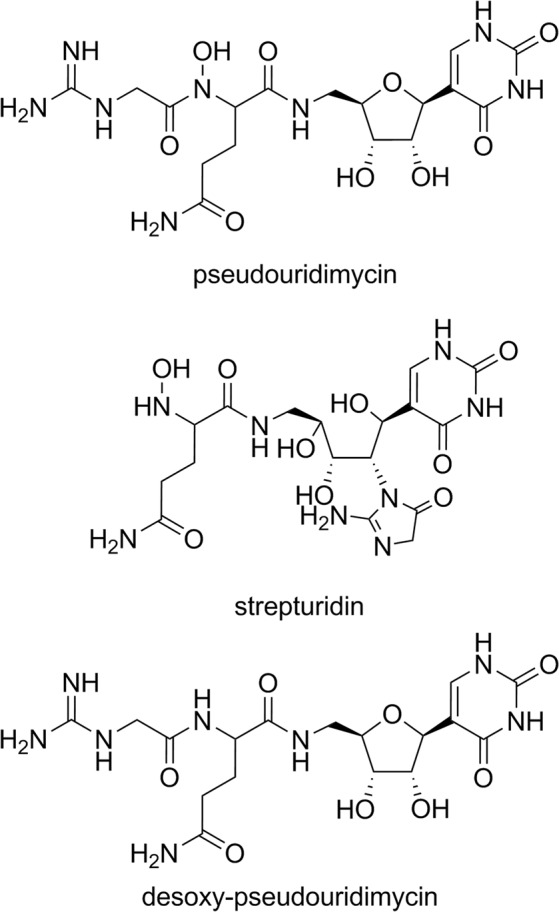
Structures of pseudouridimycin (PUM), strepturidin (STU) and desoxy-pseoudouridimycin (dPUM).

The emergence of antibiotic resistant microorganisms is currently one of the most crucial problems to be solved by pharmaceutical research. Screening of natural compounds for antibiotic activity has been utilized to find new classes of pharmaceuticals, but new discoveries have become less frequent as the most available sources have already been exhausted. However, a new promising active compound, i.e. pseudouridimycin (PUM, Fig. 1), representing a novel type of antibiotic, was recently discovered by screening 3000 actinobacterial and fungal extracts^1^. The two bacterial strains responsible for the detected activity belonged to the genus of *Streptomyces* soil bacteria. PUM is a C-nucleoside analogue that selectively inhibits bacterial RNA polymerase (RNAP) and it was found to be effective against both Gram-negative and Gram-positive bacteria^1^. As opposed to the other two classes of antibiotic bacterial RNAP inhibitors in clinical use, rifamycins and lipiarmycins, PUM targets a different site, namely the nucleotide addition site in the active center of the enzyme. This directly blocks the active site and halts transcription. Recently, the metabolic pathway responsible for formation of PUM has been identified^2^, which has shed light on the biosynthesis of C-nucleosides and provides possibilities for production of PUM analogs by metabolic engineering. Isolation and characterization of another C-nucleoside analogue, strepturidin (STU, Fig. 1) from *Streptomyces albus* DSM 40763 was reported in 2014^3^. STU shares structural similarities with PUM. Both compounds contain pseudouridine base moieties and *N*-hydroxylated glutamines bound to the 5′-amino group of a pentose carbohydrate. STU lacks the guanidinylated glycine moiety, but instead it contains an acyclic carbohydrate moiety in place of the ribose unit. However, at this time no additional studies have been reported on STU.

In the present study, C-nucleoside analogues were isolated from *Streptomyces albus* DSM 40763 in order to evaluate their potential specific inhibition of RNAPs. The two compounds extracted were identified by HRMS and NMR spectroscopy together with chemical derivatization methods and they were found to be desoxy-pseudouridimycin (dPUM) and PUM. The DSM 40763 has not been reported to produce either PUM or dPUM before. STU could not be detected from culture extracts, which is inconsistent with the previously reported^3^ findings. The spectroscopic and chemical analyses of the extracts revealed that PUM and dPUM have the same characteristics previously reported for STU and desoxygenated STU (dSTU). Genome sequencing revealed a biosynthetic gene cluster similar to the known PUM pathway. RNAP inhibition assays provided comparable activities to those reported for PUM. According to this data, the existence of STU may be questioned and the previously reported STU may, in fact, be PUM.

## Results and Discussion

### Isolation of the secondary metabolites

**Figure 2:**
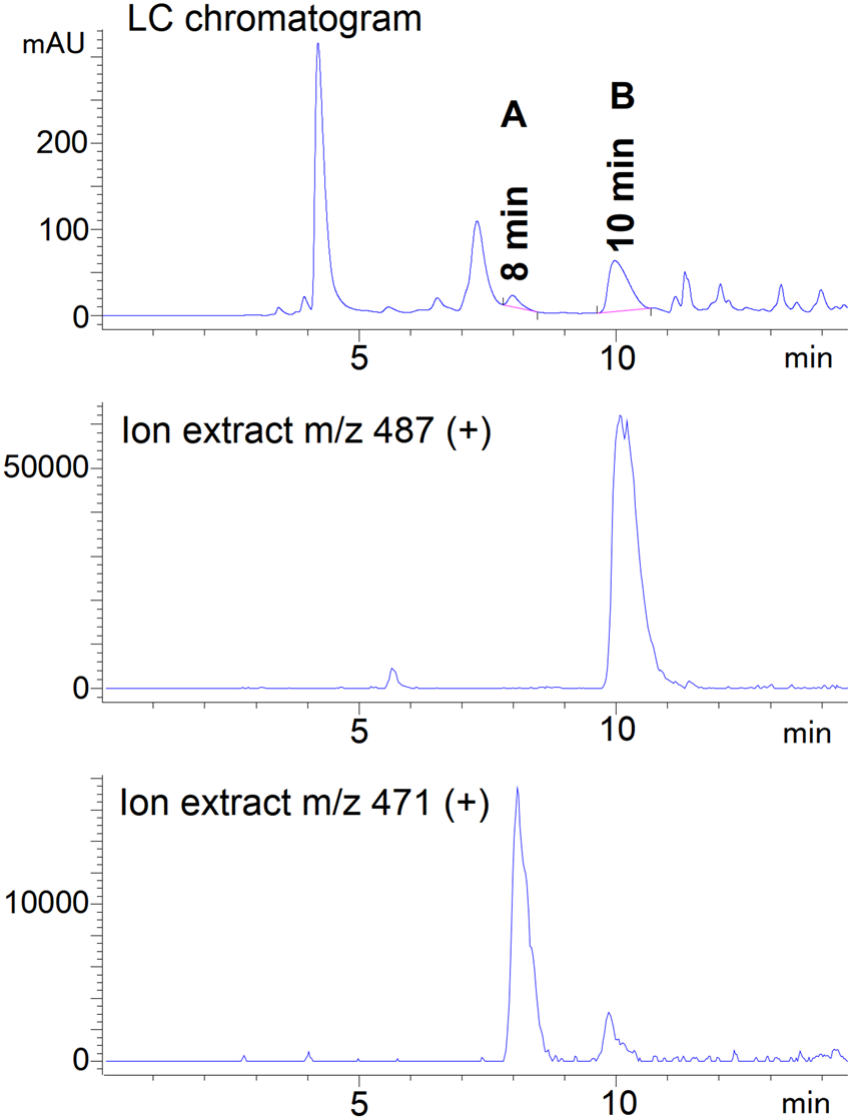
LC-MS chromatogram of the culture extract. Positive ion extracts with the shown m/z values correspond to the isolated peaks (**A**,**B**). UV detection wavelength = 260 nm.

In order to obtain C-nucleosidic secondary metabolites, *S. albus* DSM 40763 was cultivated under conditions similar to those reported for STU production and the medium extracts were screened by LC-MS. Once compounds with m/z values corresponding to PUM or STU and dPUM were detected, the strain was grown in a larger scale in a 3 l bioreactor to obtain sufficient material for structure elucidation of the metabolites. Two compounds (products **A** and **B** in Fig. 2) were observed and isolated from the culture broth using activated charcoal extraction, followed by chromatographic purifications that gave homogeneous products.

### Characterization of the isolated compounds

For the characterization of the isolated compounds ^1^H^13^,C, COSY, HSQC and HMBC NMR experiments and HRMS spectroscopic techniques were first applied. By HRMS m/z values of 487.1897 (positive mode, calcd. for C_17_H_27_N_8_O_9_^+^, 487.1896) and 469.1802 (negative mode, calcd. for C_17_H_25_N_8_O_8_^−^, 469.1801) were observed for the products. The former value corresponds to the masses calculated for STU or PUM (product **B**), both molecules having the same exact mass, and the latter m/z value refers to dPUM (product **A**). The NMR characterization (matching well to previously reported ^1^H^13^,C and 2D data) verified readily the authenticity of dPUM, but the discrimination whether the other isolated compound was STU or PUM proved to be more complex. The reported 1D NMR chemical shifts for STU and PUM^1,3^ resemble each other and direct comparison of the measured ^1^H and ^13^C NMR data could not reliably distinguish the identity of the isolated metabolite (see Tables S3 and S4 in SI for side by side comparison of the reported chemical shifts and the ones measured in the present study).

**Figure 3:**
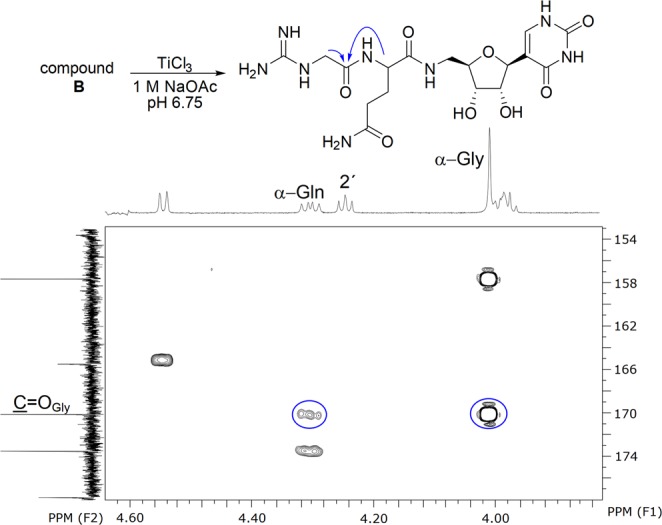
Synthetic modification of B into dPUM (compound A) and a key segment of HMBC spectrum. Blue arrows indicate the HMBC correlations corresbonding to the circled signals on the spectrum revealing the adjacency of the Gln and Gly moieties.

In D_2_O, two spin-coupled systems of protons (i.e. protons of the sugar and glutamine moieties) and two spin-isolated systems (single and two protons) were detected. The spin-isolated single proton on the low field could be assigned to the base moiety (H6, pseudouracil). HMBC measurements revealed one carbon (C1 at 110.3 ppm) that coupled to both this proton and the spin-coupled system belonging to the six protons of the sugar moiety (H1′, H2′, H3′, H4′, H5′ and H5″). The H5′ and H5″ were coupled to a carbonyl carbon (Gln C=O, 171.1 ppm) that was also coupled to the five protons of the glutamine moiety (Gln-Hα, -Hβ and -Hγ). These signals are uniform in both PUM and STU and do not yet reveal the location of the spin-isolated methylene protons. The key difference between the two compounds is whether the methylene group belongs to a glycine moiety as in the structure of PUM or to a cyclic 2-aminoimidazol-5-one as in STU. In the case of STU, HMBC correlations have previously been reported to reveal the location of the 2-aminoimidazol-5-one moiety, bound to the acyclic sugar moiety^3^. However, the signals of the 2′-proton and the methylene protons were overlapping both in D_2_O and in a mixtures of D_2_O and DMSO-*d*_6_, which complicated their spectroscopic assignment (Supplemental Material). As the 2D data proved insufficient for the characterization, the compound was subjected to chemical derivatization and exposed to a titanium trichloride treatment^1,4^, which may be used for the reduction of the *N*-hydroxy group. The reaction product was proven to be identical to the isolated dPUM by NMR spectroscopy and HPLC-coelution test (Fig. S16 in SI). In this case, the HMBC data showed readily detectable HMBC correlations in D_2_O between the carbonyl carbon (Gly C=O) and alpha protons of both the Gln and Gly moieties (Fig. 3). Thus the isolated compound (**B**) could be identified as PUM.

Tandem mass spectroscopy with PUM and dPUM (Fig. S15 in SI) showed fragmentation patterns similar to those reported for STU and Gln-STU^3^ which supports the assumption that PUM is reassigned STU.

### Isolated product B inhibits transcription by multisubunit RNAPs by competing with UTP

**Figure 4:**
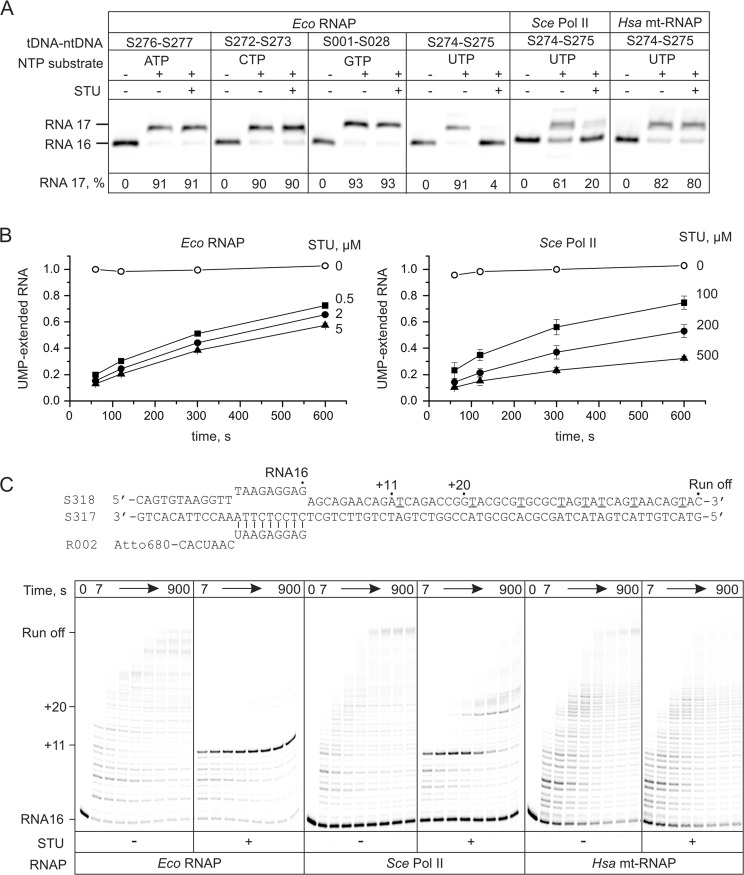
The effects of the isolated product (B) on transcription by *E. coli* (*Eco*), *S. cerevisiae* (*Sce*) Pol II and human (*Hsa*) mitochondrial RNA polymerases. The oligonucleotides used for assembling the TECs are shown in Supplementary Fig. S1. (**A**) Single nucleotide incorporation assay performed for 2 min at 5 μM NTP substrates in the presence and absence of 100 μM of B. The fraction of extended RNA is indicated below each gel lane. (**B**) Time courses of UMP incorporation by *Eco* RNAP (left graph) and *Sce* Pol II (right graph) at 5 μM UTP in the presence of the indicated concentrations of STU. Error bars indicate the range of duplicate measurements. (**C**) The effect of B on processive transcript elongation. The schematic of the nucleic acid scaffold used for assembly of the transcript elongation complexes is presented above the gel panels, thymidines in the non-template strand are underlined and correspond to uridines in the nascent RNA transcript. The transcript positions are numbered relative to the 3′ end of the RNA primer with position +1 corresponding to the first incorporated nucleotide. Positions corresponding to strong pauses induced by B are indicated on the left from gel panels. An independent repeat of experiments in (**A**,**C**) is presented in the Supplementary Fig. S2.

We first tested the effect of the isolated product (**B**) in a single nucleotide addition assay performed at 5 μM NTP substrates for 2 minutes. High concentration of **B** (100 μM) did not measurably inhibit incorporation of AMP, GMP and CMP, but strongly delayed incorporation of UMP into the nascent RNA by *E. coli* (*Eco*) RNAP (Fig. 4A). Incorporation of UMP into the nascent RNA by *S. cerevisiae* RNA polymerase II (*Sce* Pol II) was also strongly inhibited, whereas no inhibition was observed in the case of human mitochondrial RNAP (*Hsa* mt-RNAP). Time courses of UMP incorporation at several concentrations of **B** revealed that *Sce* Pol II required approximately 100-fold higher concentration of **B** than *Eco* RNAP to achieve a similar magnitude of the inhibition (Fig. 4B). We then investigated the effect of 100 μM **B** on the processive transcription through 49 base pairs of the template DNA at 5 μM NTP substrates (Fig. 4C). Transcription by *Eco* RNAP was nearly completely halted at +11, one base pair upstream of the incorporation of the first UMP into the nascent RNA. *Sce* Pol II also strongly paused at +11, but cleared the pause within the timeframe of the experiment (15 min) and paused again at +20, just before incorporation of the second UMP. At the same time, processive transcript elongation by *Hsa* mt-RNAP was not affected by 100 μM of **B**. Overall, the above experiments demonstrate that the isolated product (**B**) possesses characteristic biological activities of PUM: it inhibits transcription by multisubunit RNAPs by competing with UTP and displays high selectivity towards the bacterial RNAP^1^.

### Identification and analysis of the *sap* (*S*treptomyces *a*lbus *p*seudouridimycin) gene cluster

**Figure 5:**
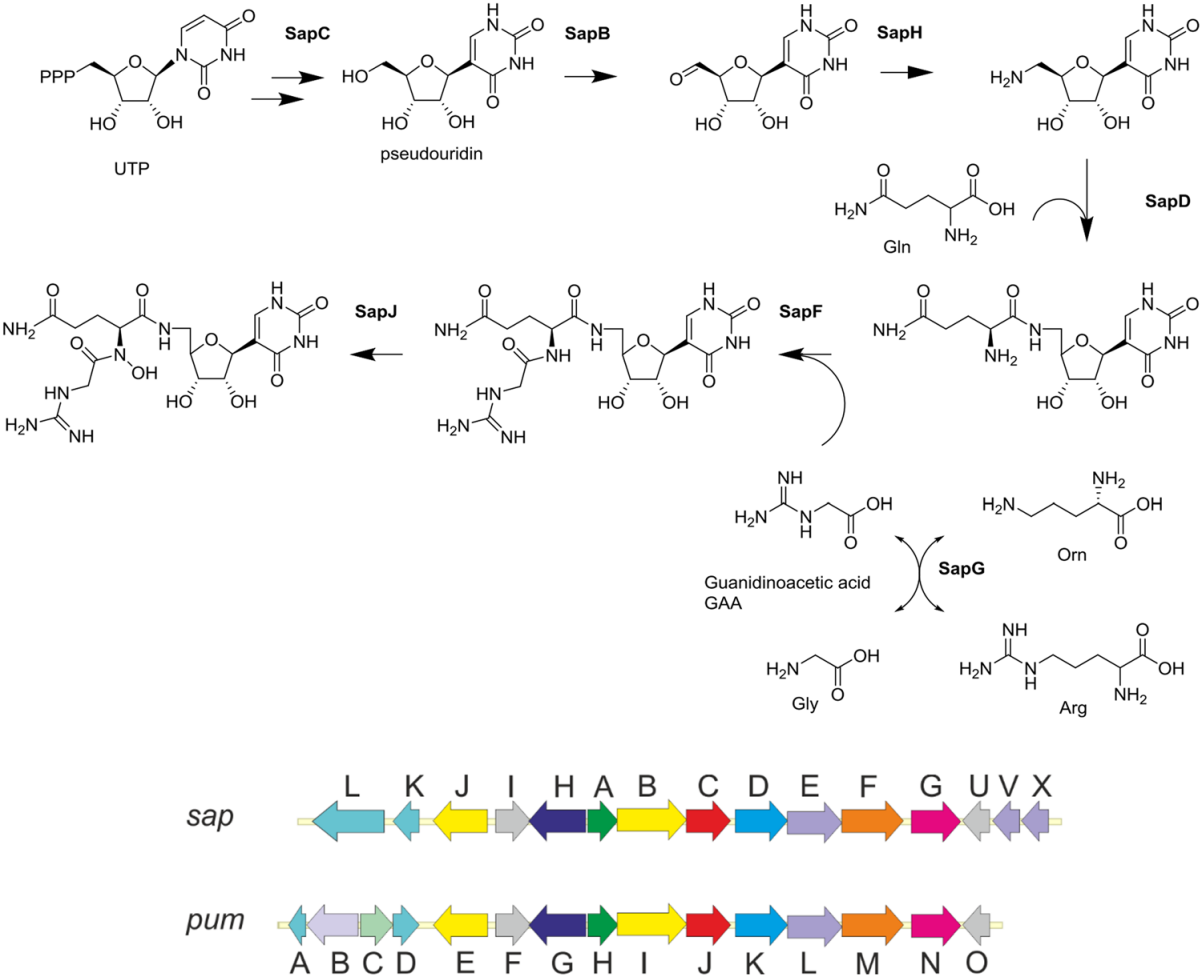
Biosynthetic pathway of pseudouridimycin and *sap* biosynthesis cluster. (**a**) Proposed biosynthetic pathway of pseudouridimycin. (**b**) Arrangement of *sap* and *pum* biosynthesis clusters.

The draft genome of *S. albus* DSM 40763 was acquired by MiSeq sequencing, which resulted in 4,517,426 reads (error corrected and trimmed down to 4,335,036) that were assembled *de novo* into 162 contigs (Supplementary Table S1). Bioinformatic analysis revealed a gene cluster containing open reading frames homologous to pseudouridine synthases and glycine amidinotransferases, which have recently been implicated in the biosynthesis of C-nucleosides^2^. The *sap* gene cluster displayed strong synteny to the *pum* pathway with the arrangement of genes from *sap*J to *sap*U (*pum*E to *pum*O) conserved (Fig. 5b) and all core genes implicated in the formation PUM could be identified. The sequence identity between the corresponding *sap* and *pum* gene products varied from 62% to 88% (Table 1). Similar gene clusters to the *sap* pathway appeared to be widely spread in *Streptomyces* and a Blast search revealed eight strains harboring over 99% identity at nucleotide level (List S1 in Supplement). However, to date only two strains, *Streptomyces* sp. ID38640 and ID38673^1^, have been reported to produce PUM in addition to *S. albus* DSM 40763.

**Table 1:** Deduced functions of genes in the *sap* cluster detected in *S. albus* DSM 40763 genome and comparison with genes in *pum* biosynthesis cluster from *Streptomyces* sp. strain ID38640.

*S. albus* DSM 40763	Size^a^	Deduced function	*Streptomyces* sp. strain ID38640	Identity/similarity/gaps (%)
CDS			CDS	
sapL	686	Peptidase	—	—
sapK	265	Hydro-lyase	—	—
sapJ	413	Oxidoreductase	pumE	81/88/0
sapI	288	Hypothetical protein	pumF	62/69/6
sapH	460	Aminotransferase	pumG	74/82/2
sapA	213	Adenylate kinase	pumH	65/75/0
sapB	510	Oxidoreductase	pumI	70/79/0
sapC	346	tRNA pseudouridine synthase	pumJ	75/80/2
sapD	395	Carboxylate amine ligase	pumK	67/75/1
sapE	426	Transporter	pumL	74/80/3
sapF	464	Carboxylate amine ligase	pumM	72/79/0
sapG	385	Amidinotransferase	pumN	83/88/0
sapU	204	Hypothetical protein	pumO	71/76/1
sapV	216	Transporter	—	—
sapX	237	Transporter	—	—

^a^Number of amino acids.

The key enzyme in the biosynthesis of PUM is likely to be SapC, which according to sequence analysis belongs to the TruD family of tRNA pseudouridine synthases. These enzymes are responsible of modification of pseudouridine^13^ in tRNA^Glu ^^5^. Pseudouridine synthases typically use RNA as substrate, but in the biosynthesis of PUM a more likely substrate for SapC is a derivative of uridine obtained from the cellular pool. SapA, a hypothetical adenylate kinase, is possibly involved in providing a phosphorylated substrate for SapC, but the identity of this compound remains to be solved. Modification of pseudouridin most likely continues by the action of SapB, which resembles dehydrogenases belonging to the glucose-methanol-choline oxidoreductase family, and SapH, a pyridoxal phosphate-dependent aminotransferase, that are responsible for the generation of 5′-oxo-pseudouiridine and 5′-aminopseudouridine, respectively. SapD and SapF are similar to ATP-grasp domain containing carboxylate-amine ligases. The established functions of the orthologues in *pum* cluster, PumK and PumM, are attachment of glutamine to aminopseudouridine and guanidinoacetic (GAA) to glutaminyl-5′-N-pseudouridine, respectively. SapG resembles proteins of amidinotransferase superfamily that contains glycine and inosamine amidinotransferases. Glycine amidinotransferases generate GAA from arginine and glycine and thereby SapG would be responsible of providing GAA substrate for SapF. SapJ is a FAD dependent oxidoreductase and is 39% identical to KijD3, an *N*-oxygenase in the biosynthesis of antibiotic kijanimicin^6^ and therefore deduced to carry out the hydroxylation of dPUM.

In addition, two hypothetical genes of unknown function, coding for SapI and SapU, were found located in analogous positions to their counterparts PumF and PumO, respectively. Altogether three transporters were encoded in the gene cluster: SapE is a Major Facilitator Superfamily protein and two ABC-transporters, SapV and SapX, reside at the right edge of the cluster. Two genes, *sapK* and *sapL*, are located on the left edge of the analyzed area that are homologous to hydro-lyases and peptidases, respectively. No orthologues to SapK and SapL are found in the *pum* cluster suggesting that they do not have a role in the biosynthesis of PUM.

## Conclusions

Here we have described the isolation and characterization of two C-nucleoside analogues produced by *Streptomyces albus* DSM 40763, which were identified unambiguously as pseudouridimycin (PUM) and desoxy-pseurouridimycin (dPUM). This is in contrast to previous studies, which have suggested that the strain produces the structurally related strepturidin (STU)^3^. The characterization of PUM was conclusive after chemical reduction of the *N*-hydroxy group yielding a product that was identical to the isolated dPUM. For dPUM, the 2D HMBC NMR experiment was of sufficient quality and revealed the locations of each spin system. The RNAP inhibition assays done with the isolated compound also provided similar activities to those measured for PUM previously^1^. Thus these observations (NMR, chemical derivatization and RNAPs inhibition data) altogether confirm the identity of the isolated compound as PUM.

We were not able to detect STU from culture extracts of *Streptomyces albus* DSM 40763 during the course of our study, but this does not formally exclude the possibility that the strain is able to synthesize both PUM and STU. The NMR data of PUM revealed similarities hardly discriminated from that previously reported for STU. Moreover, the MS/MS-data of PUM and dPUM showed the same daughter ions and similar fragmentation patterns previously reported for STU and dSTU. Genome sequencing revealed a cluster containing genes highly similar to those with established functions found in the *pum* cluster^2^. A key differences in the biosynthesis of these two metabolites would be in the formation of the cyclic 2-aminoimidazol-5-one moiety from guanidine acetic acid in STU, but the *sap* gene cluster does not encode any proteins that could feasibly catalyze the cyclization reaction. Another critical difference is in the regiochemistry of the attachment of these two units, but the high sequence identity of 72% between the two GAA transferases (*sapF* and *pumM*) suggests that the genes are orthologous and responsible for PUM formation. The characteristics of PUM and dPUM proved to be the same previously reported for STU and dSTU. Hence, the existence of STU may be questioned and STU, in fact, is PUM.

## Methods

### Reagents and oligonucleotides

HPLC purified DNA oligonucleotides were purchased from Eurofins Genomics GmbH (Ebersberg, Germany). PAGE purified ATTO-680 labeled RNA primer was purchased from IBA Biotech (Göttingen, Germany). NTPs were from Jena Bioscience (Jena, Germany). DNA oligonucleotides and RNA primers are presented in Supplementary Fig. S1. All other reagents used were molecular biology grade.

### General remarks

The NMR spectra were recorded with a Bruker Avance 500 MHz spectrometer. The high resolution mass spectra were recorded with a Bruker Daltonics microTOF-Q instrument. The LC-MS chromatograms were recorded with a Agilent Technologies 1260 Infinity Binary LC and Agilent 6100 Series Quadrupole LC/MS Systems with Phenomemex 150 × 4.6 Synergi^TM^ 4 μm Fusion-RP 80 Å analytical column (flow rate 0.5 ml/min and wavelength 260 nm, 0.1% formic acid in H_2_O and MeCN as eluents).

#### Production and isolation of the secondary metabolites

Cultivations of *S. albus* DSM 40763 were performed using media described for production of STU^3^ with minor modifications. Precultures were cultivated in 50 ml of media containing 1% mannitol, 0.5% Bacto^TM^ peptone, 0.5% yeast extract and 0.092% CaCl_2_ * 2 H_2_O in 250-ml Erlenmeyer flasks for 2 days. Production of secondary metabolites was performed in three-liter volume in Fermentec FMT ST Series bioreactors using 5% inoculum and medium containing 1% mannitol, 1% soy meal, 0.6% Nutrient Broth (Biokar Diagnostics), 0.1% CaCO_3_ and 0.1% polypropylene glycol P 2,000 as antifoam. Temperature was set to 30 °C, stirring to 300 r.p.m., and aeration to 1 v.v.m. (volume per volume per minute). After 2–3 days of fermentation the broth was collected and cells were removed by centrifugation. To the remaining broth activated charcoal, 2 g l^−1^ was added and the mixture was stirred overnight at 4 °C, after which the supernatant was removed by centrifugation and subsequent filtration. The collected charcoal was placed in a Büchner funnel and eluted with copious amounts of a 1:1 mixture of H_2_O and acetone to extract the compound. The extracts were concentrated to a small volume and then fractioned on a reverse phase silica column eluting with a mixture of H_2_O and MeCN (95:5). The fractions containing the compounds of interest were combined and evaporated to a small volume. Finally, PUM was isolated by a RP-HPLC (Kinetex 250 × 10, 5 μm C-18 LC-column 100 Å, flow rate 3 ml/min) eluting with a gradient of 25 mM triethylammonium acetate buffer in H_2_O and MeCN, and subsequently desalted by eluting the collected fractions with pure H_2_O and MeCN. Collected fractions were freeze-dried to yield a white solid (19 mg, 39 μmol). ^1^H NMR (500 MHz, D_2_O): *δ* 7.53 (s, 1H, H6), 4.92 (dd, 1H, *J* = 6.5 & 6.0 Hz, Hα of glutamine), 4.56 (d, 1H, *J* = 5.5 Hz, H-1′), 4.29 and 4.23 (2 × d, 2H, *J* = 15.5 Hz, both, Hα of Gly), 4.22 (m, 1H, H-2′), 4.01–3.97 (m, 2H, H-3′ and H-4′), 3.51 (dd, 1H, *J* = 12.0 & 4.0 Hz, H-5′), 3.45 (dd, 1H, *J* = 11.5 & 3.0 Hz, H-5″), 2.30 (m, 2H, Hγ of glutamine), 2.16 (m, 2H, Hβ of glutamine); ^13^C NMR (125 MHz, D_2_O): *δ* 178.0 (C-δ of glutamine), 171.1 (C=O of glutamine), 171.0 (C=O of glycine), 165.1 (C-2), 157.5 (guanidyl), 152.8 (C-4), 141.6 (C-6), 110.3 (C-1), 81.1 (C-4′), 79.7 (C-1′), 73.1 (C-2′), 72.0 (C-3′), 59.7 (C-α of glutamine), 42.5 (C-α of glycine), 41.1 (C-5′), 31.2 (C-γ of glutamine), 23.2 (C-β of glutamine). HRMS (ESI) *m/z*: [M + H]^+^ calcd for C_17_H_27_N_8_O_9_^+^ 487.1896; found 487.1897.

dPUM was isolated using methods mentioned above yielding a white solid (6.6 mg, 14.0 μmol). ^1^H NMR (500 MHz, D_2_O): *δ* 7.54 (s, 1H, H-6), 4.55 (d, 1H, *J* = 5.9 Hz, H-1′), 4.30 (dd,, 1H, *J* = 5.5 & 8.9 Hz, H-α of glutamine), 4.25 (dd, 1H, *J* = 5.5 & 5.0 Hz, H-2′), 4.01 (s, 2 H, H-α of glycine), 3.96–4.00 (m, 2H, H-3′ & H-4′), 3.53 (dd, 1H, *J* = 14.5 & 5.5 Hz, H-5′), 3.43 (dd, 1H, *J* = 14.5 & 3.5 Hz, H-5″), 2.32 (dd, *J* = 7.5 Hz, both, 2H, H-γ of glutamine), 1.91–2.09 (m, 2H, H-β of glutamine); ^13^C NMR (125 MHz, D_2_O): *δ* 177.8 (C-δ of glutamine), 173.5 (C=O of glutamine), 170.1 (C=O of glycine), 165.1 (C-2), 157.5 (guanidyl), 152.8 (C-4), 141.8 (C-6), 110.1 (C-1), 81.3 (C-4′), 79.7 (C-1′), 72.9 (C-2′), 71.9 (C-3′), 53.6 (C-α of glutamine), 43.6 (C-α of glycine), 41.1 (C-5′), 31.1 (C-γ of glutamine), 26.9 (C-β of glutamine). HRMS (ESI) *m/z*: [M−H]^−^ calcd for C_17_H_25_N_8_O_8_^−^; 469.1801 found 469.1802.

#### Reduction of the *N*-hydroxy group

Solution of PUM (2.5 mg, 5.1 μmol) in 1 M sodium acetate buffer (pH 6.75) (40 μl) was treated with TiCl_3_ (1.6 mg, 10.4 μmol) and the reaction was allowed to proceed for 2 hours at room temperature (the reaction was completed according to LC-MS analysis). The reaction mixture was diluted with water (0.5 ml), filtered, and the product was isolated without further work up by RP-HPLC (Phenomemex 250 × 10 Synergi^TM^ 4 μm Fusion-RP 80 Å, flow rate 3 ml min^−1^), eluting with a gradient of H_2_O and MeCN. The collected fractions were freeze-dried to yield dPUM as a white solid (1.0 mg, 2.1 μmol, 41%). The spectral data was identical to that measured for isolated dPUM.

#### Proteins

*E. coli* RNAP was expressed in *E. coli* Xjb(DE3) (Zymo Research, Irvine, CA, USA) bearing pVS10 plasmid and purified by Ni-, heparin and Q-sepharose chromatography as described previously^7^, dialyzed against the Storage Buffer (50% glycerol, 20 mM Tris-HCl pH 7.9, 150 mM NaCl, 0.1 mM EDTA, 0.1 mM DTT) and stored at −20 °C. *S. cerevisiae* RNA polymerase II was purified from *S. cerevisiae* strain SHy808 (kindly provided by the laboratory of Mikhail Kashlev, NIH, National Cancer Institute, Frederick, MD, USA) largely as described previously^8,9^. Human mitochondrial RNA polymerase lacking 213 N-terminal amino acids (mitochondrial localization signal and an unstructured regulatory domain) was expressed in *E. coli* as follows. Plasmid pGB163 (*T7 promoter*-His_6_-Δ213*mtRNAP*) was transformed into *E. coli* T7 Express lysY/I^q^ cells from New England Biolabs (Ipswich, MA, USA). Cells were grown in 1 L LB medium supplemented with 50 μg/ml kanamycin at 37 °C until OD 0.6, the culture was transferred to 25 °C, and protein expression was induced for 5 h by the addition of 0.8 mM IPTG. Cells were harvested by centrifugation at 6,000 × g, 4 °C for 10 min, resuspended in Lysis Buffer (50 mM Tris-HCl pH 6.9, 500 mM NaCl, 5% glycerol) supplemented with 1 mM β-ME, a tablet of EDTA-free protease inhibitors (Roche Applied Science, Penzberg, Germany), 1 mg/ml lysozyme, incubate on ice for 30 min and disrupted by sonication. The lysate was cleared by centrifugation at 18,000 × g, 4 °C for 30 min. The supernatant was supplemented with 10 mM imidazole and loaded onto Ni-sepharose (GE Healthcare, Chicago, IL, USA) column pre-equilibrated with Lysis Buffer. Protein was eluted using a strep gradient (20, 50, 250 mM) of imidazole in lysis buffer. The 250 mM imidazole fraction containing RNAP was further purified using Heparin and Resource-S column in Buffer A (50 mM Tris-HCl pH 6.9, 5% glycerol, 1 mM β-mercaptoethanol, 0.1 mM EDTA) and Buffer B (Buffer A supplemented with 1.5 M NaCl). Δ213mtRNAP eluted at ≥ 50 and ≥ 30% Buffer B from Heparin and Resource-S columns, respectively. The fractions containing the purified protein were concentrated using Amicon Ultra-4 centrifugal filters (Merck Milipore, Burlington, MA, USA), dialyzed overnight in Storage Buffer (10 mM Tris-HCl pH 7.5, 50% glycerol, 100 mM NaCl, 0.1 mM EDTA, 0.1 mM DTT) and stored at −80 °C. Plasmids used for protein expression are listed in Supplementary Table S2.

#### TEC assembly

TECs (1 μM) were assembled by a procedure developed by Komissarova *et al*.^10^. The assembly was carried out in TB10 buffer (40 mM HEPES-KOH pH 7.5, 80 mM KCl, 10 mM MgCl_2_, 5% glycerol, 0.1 mM EDTA, and 0.1 mM DTT). An RNA primer (final 1 μM) was annealed to the template DNA (final 1.4 μM), incubated with RNAP (1.5 μM) for 10 min, and then with the non-template DNA (2 μM) for 20 min at 25 °C. The nucleic acid scaffolds used for assembling the TECs are presented in Supplementary Fig. 1.

#### *In vitro* transcription reactions, single nucleotide addition assay

The transcription reactions were initiated by the addition of 5 μM NTP to 0.1 μM TEC in TB10 buffer (total final volume 20 μl) pre-incubated with the indicated concentrations of compound **B** for 2 minutes, samples were incubated for the indicated time intervals at 25 °C, and the reactions were stopped by the addition of 30 μl of Gel Loading Buffer (94% formamide, 20 mM Li_4_-EDTA and 0.2% Orange G). RNAs were separated on 16% urea-PAGE gel and visualized with Odyssey Infrared Imager (Li-Cor Biosciences, Lincoln, NE, USA); band intensities were quantified using ImageJ software^11^.

#### *In vitro* transcription reactions, processive transcript elongation

The transcription reactions were initiated by the addition of 5 μM NTP with or without **B** (100 μM final concentration) to 0.5 μM TEC in TB10 buffer at 25 °C. 10 μl aliquots were withdrawn at the indicated time points and quenched with 30 μl of Gel Loading Buffer. RNAs were separated on 16% urea-PAGE gel and visualized with Odyssey Infrared Imager (Li-Cor Biosciences).

#### Isolation of genomic DNA and genome sequencing

*Streptomyces albus* DSM40763 was cultured in 30 ml of GYM media with 0.5% glycine at 30 °C for 2 days shaking at 300 rpm. The cells were pelleted and frozen at −20 °C for 4 days. Genomic DNA was extracted using the protocol developed by Nikodinovic *et al*.^12^ with slight modifications. Quality control and the PCR-free shotgun library (Illumina) was prepared at Eurofins Scientific (Ebersberg, Germany). A single lane of an Illumina MiSeq v3 sequencer was used to produce 2 × 300 bp reads.

The quality of the reads were manually checked before and after error correction using FASTQC (v0.11.2)^13^. The reads were assembled using A5-miseq (v20150522)^14^, contiguated with ABACAS (v1.3.1)^15^ using *Streptomyces albus* NK660 (CP007574.1) as the reference, and the gaps were filled using IMAGE (v2.4.1)^16^. The final assembly was annotated using RAST^17^ and evaluated for completeness using BUSCO (v1.22)^18^. All programs were used with the default parameters and run on the CSC – IT Center for Science’s Taito super-cluster (Espoo, Finland). This Whole Genome Shotgun project has been deposited at DDBJ/ENA/GenBank under the accession RCIY00000000. The version described in this paper is version RCIY01000000.

## Supplementary information

Supporting information
