# Supplementary material for: Characterization of C-nucleoside Antimicrobials from Streptomyces albus DSM 40763: Strepturidin is Pseudouridimycin
Source: Sci Rep. 2019 Jun 20;9:8935. doi: 10.1038/s41598-019-45375-w (PMC6586884; doi:10.1038/s41598-019-45375-w)
Supplement: Supplementary file 1 — Supporting information [file 41598_2019_45375_MOESM1_ESM.docx]

**Characterization of C-nucleoside Antimicrobials from *Streptomyces albus* DSM 40763: Strepturidin or Pseudouridimycin**

Petja Rosenqvist^a^, Kaisa Palmu^b^, Ranjit Kumar Prajapati^b^, Keith Yamada^b^, Jarmo Niemi^b,^ Georgiy A. Belogurov^b^, Mikko Metsä-Ketelä^b^, Pasi Virta^a^

^a^Department of Chemistry, University of Turku, FIN-20014 Turku, Finland

^b^Department of Biochemistry, University of Turku, FIN-20014 Turku, Finland

**Supplementary Information**

**Table of Contents**

**S2** Nucleic acids scaffolds used to assemble TECs

**S3** The eﬀects of **B** on transcription, an independent repeat of experiments in the main text Figure 3.

**S3** List of strains with gene clusters identical to *sap* cluster.

**S4** Assembly statistics table.

**S4** Table with the plasmids in protein expression

**S5-S7** Copies of NMR spectra of isolated PUM

**S7-S9** Copies of NMR spectra of semisynthetic dPUM

**S10-S11** Copies of high resolution mass spectra of PUM and dPUM.

**S12** MS/MS spectra of PUM and dPUM

**S13** HPLC-traces of isolated and semisynthetic dPUM

**S13** Table of ^1^H-NMR data reported and measured here for PUM/STU

**S14** Table of ^13^C-NMR data reported and measured here for PUM/STU


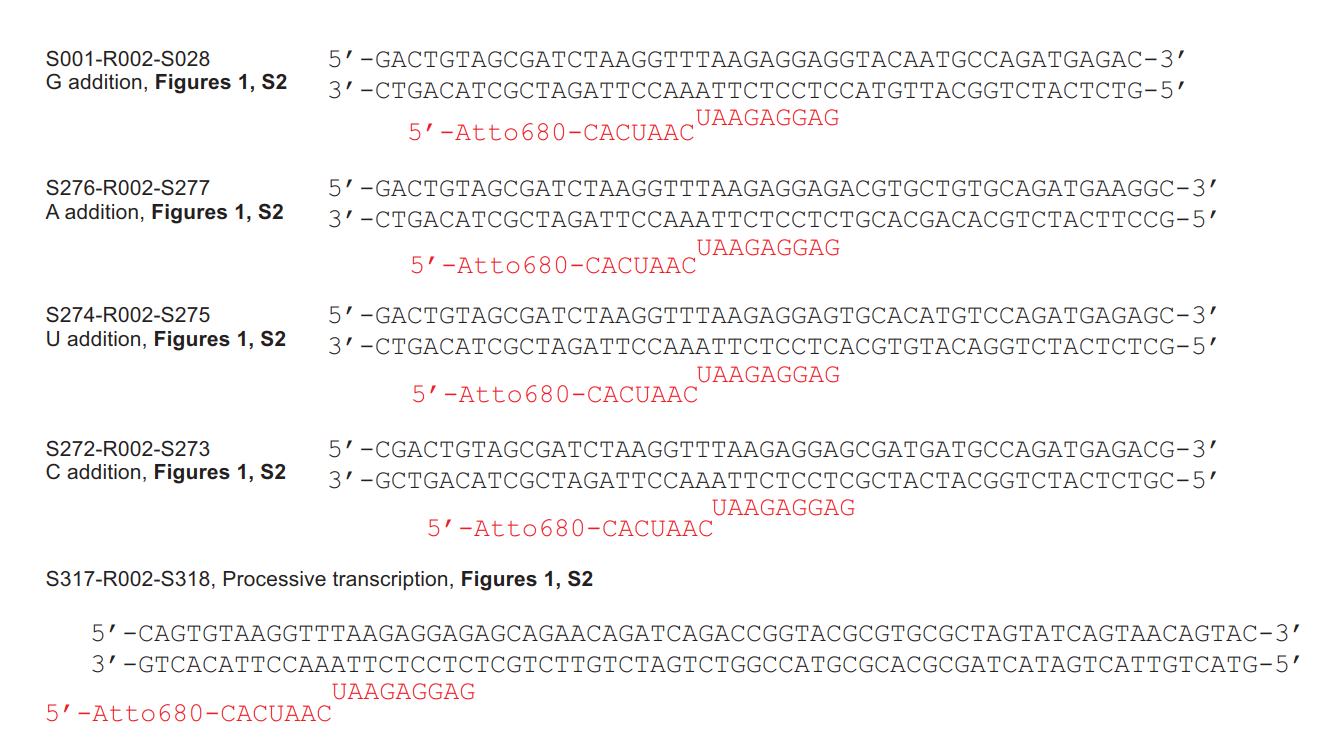


**Figure S1.** Nucleic acids scaﬀold used to assemble TECs employed in this work. The identiﬁers for oligonucleotides (internal naming used in the laboratory, tDNA-RNA-ntDNA) used for scaﬀold assembly are indicated to the left of schematics.


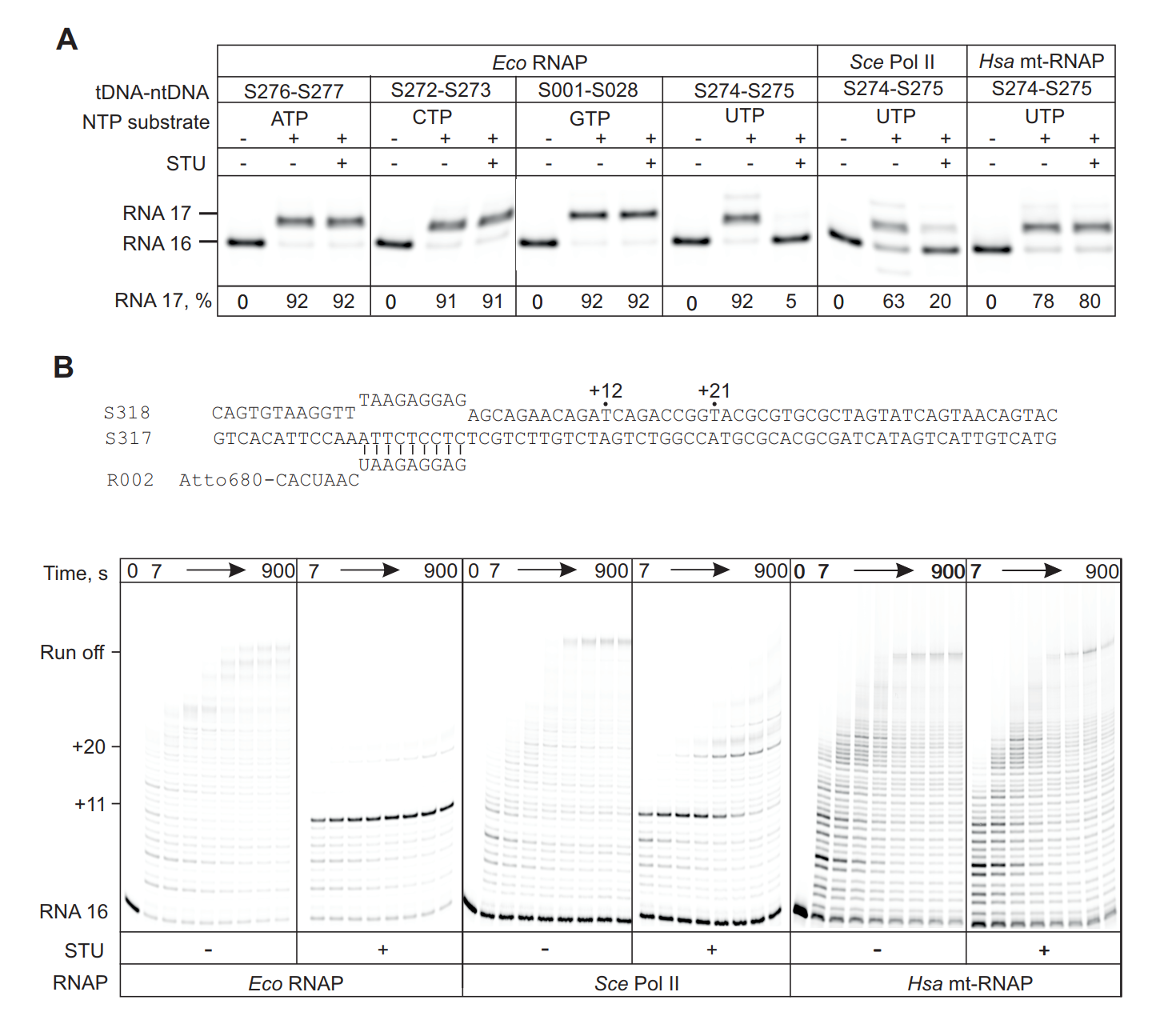


**Figure S2.** The eﬀects of compound **B** on transcription by E. coli (Eco), S. cerevisiae (Sce) Pol II and human (Hsa) mitochondrial RNA polymerases. An independent repeat of experiments in the main text Figure 4. (A) Single nucleotide incorporation assay performed for 2 min at 5 μM NTP substrates in the presence and absence of 100 mM of **B**. The fraction of extended RNA is indicated below each gel lane. The oligonucleotides used for assembling the TECs are shown in Supplementary Figure 1. (B) The eﬀect of **B** on processive transcript elongation. The schematic of the nucleic acid scaﬀold used for assembly of the transcript elongation complexes is presented above the gel panels, thymidines in the non-template strand are underlined and correspond to uridines in the nascent RNA transcript. The transcript positions are numbered relative to the 3’ end of the RNA primer with position +1 corresponding to the ﬁrst incorporated nucleotide. Positions corresponding to strong pauses induced by **B** are indicated on the left from gel panels.

**List S1.** List of strains with gene clusters identical to *sap* cluster.

*Streptomyces* sp. NRRL F-6602, *Streptomyces* sp. HPH0547, *Streptomyces graminis* strain NRRL B-1570, *Streptomyces rangoonensis* strain NRRL B-12378, *Streptomyces albus* strain NBRC 13014, *Streptomyces albus* subsp. *albus* strain NRRL B-1811, *Streptomyces almquistii* strain NRRL B-1685, *Streptomyces gibsonii* strain NRRL B-1335.

**Table S1.** Assembly statistics. ABACAS ordered and aligned the contigs into 121 scaffolds with an N50 of 488,914. The final genome assembly is 8.1 Mbp with a GC content of 72.6% and median coverage of 100x. The BUSCO analysis searched for 40 single-copy orthologs and found 38 (95%) were complete. Out of the 38 complete BUSCOs, 4 were found multiple times throughout the assembly. Futhermore, one BUSCO was identified as fragmented.

| Genome size | 8,099,623 |
| --- | --- |
| Percent GC | 72.6 |
| N50 | 488,914 |
| Longest contig length | 804,804 |
| Contigs | 162 |
| Scaffolds | 121 |
| Median coverage | 100 |
|  |  |
| Complete single-copy BUSCOs | 34 |
| Complete multi-copy BUSCOs | 4 |
| Fragmented BUSCOs | 1 |
| Missing BUSCOs | 0 |

**Table S2**.

| Strains/plasmid | Characteristics | Source/references |
| --- | --- | --- |
|  |  |  |
| Strains |  |  |
|  |  |  |
| *E.coli* |  |  |
| Xjb(DE3) |  | Zymo Research |
| T7 Express lysY/I^q^ |  | New England Biolabs |
|  |  |  |
| *S. cerevisiae* |  |  |
| SHy808 | His_6_Rpb3 | Kireeva *et al.*^a^ |
|  |  |  |
| Plasmid |  |  |
| pVS10 | T7 *promoter* -α-β-β′His_6_-ω | Svetlov *et al*.^b^ |
| pGB163 | *T7 promoter*-His_6_∆213*mtRNAP* | This study |

**a.** Kireeva, M. L. *et al.* *Methods Enzym* **370**, 138–155 (2003). **b**. Svetlov, V. & Artsimovitch, I. *Methods Mol Biol* **1276**, 13–29 (2015).

**Copies of NMR spectra of isolated pseudouridimycin**

H1´

Gln β

Gln γ

Gly α

Gln α

H5´

H5´´

H4´

H3´

H2´

H6

**Figure S3.** 500 MHz (D_2_O) ^1^H NMR spectrum of pseudouridimycin.

PPM

160

140

120

100

80

60

40

20

0

Gln α

Gly α

Gln β

Gln γ

**Figure S4.** 125 MHz (D_2_O) ^13^C NMR spectrum of pseudouridimycin.

C2´

C4´

C1´

guanidyl

Gly C=O

Gln C=O

C2

Gln δ

C5´

C3´

C4

C6

C1

**Figure S5.** ^1^H,^1^H-COSY spectrum of pseudouridimycin.

**Figure S6.** ^1^H,^13^C-HMBC spectrum of pseudouridimycin.

**Figure S7.** ^1^H,^13^C-HSQC spectrum of pseudouridimycin.

**Copies of NMR spectra of semisynthetic desoxy-pseudouridimycin**

Gln α

Gln γ

Gln β

H5´

H5´´

H4´

H3´

Gly α

H2´

H1´

H6

**Figure S8.** 500 MHz (D_2_O) ^1^H NMR spectrum of desoxy-pseudouridimycin.

C4´

C2´

Gln β

Gly α

Gln α

C3´

Gln γ

C1´

C4

Gly C=O

C5´

Gln C=O

guanidyl

Gln δ

C2

C1

C6

C4

**Figure S9.** 125 MHz (D_2_O) ^13^C NMR spectrum of desoxy-pseudouridimycin.

**Figure S10.** ^1^H,^1^H-COSY spectrum of desoxy-pseudouridimycin.

**Figure S11.** ^1^H,^13^C-HMBC spectrum of desoxy-pseudouridimycin.

**Figure S12.** ^1^H,^13^C-HSQC spectrum of desoxy-pseudouridimycin.

**Copies of mass spectra of PUM and dPUM.**

**Figure S13.** Positive mode high resolution mass spectrum of pseudouridimycin.

**Figure S14.** Negative mode high resolution mass spectrum of desoxy-pseudouridimycin.


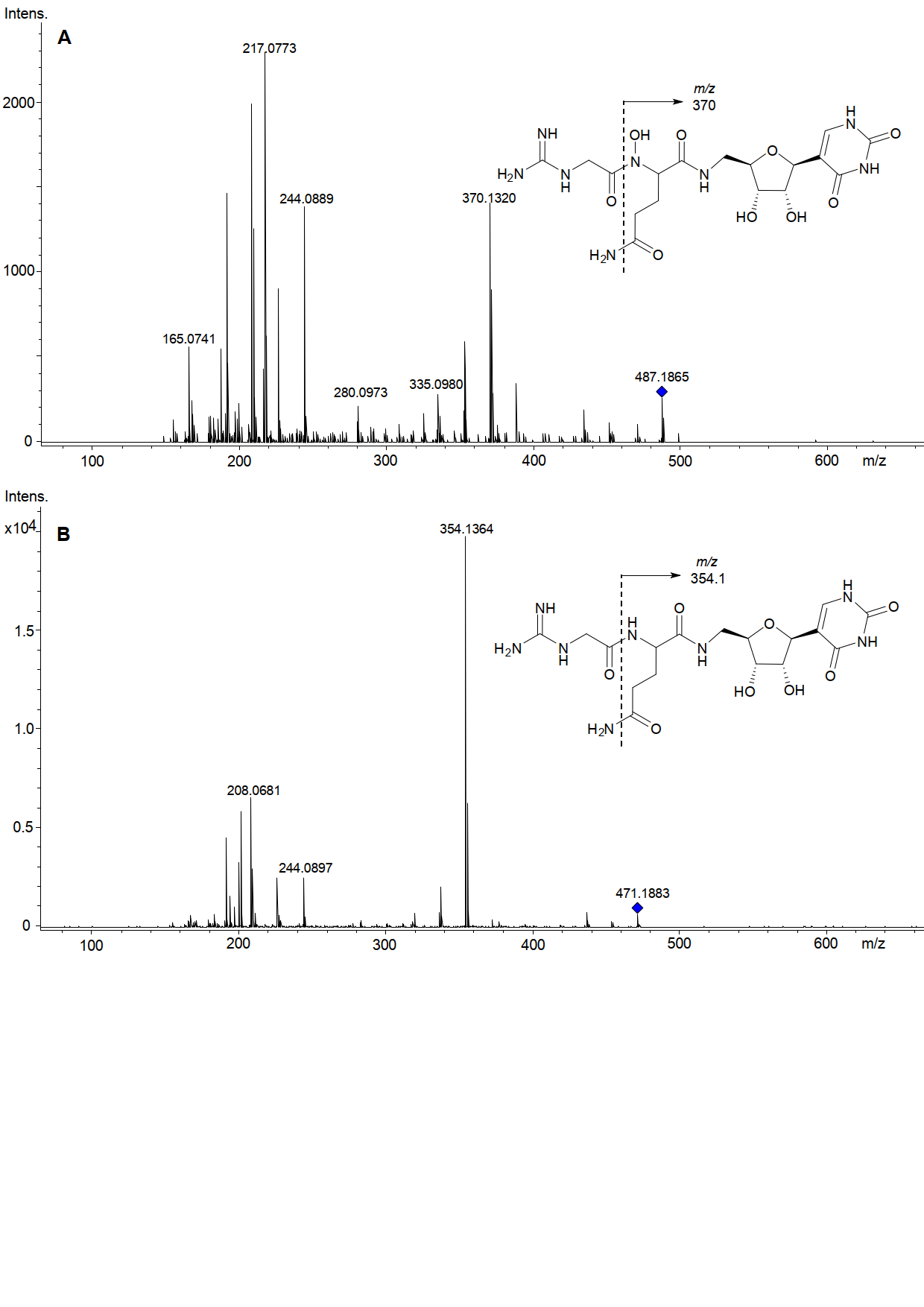


**Figure S15.** (+)-MS^2^-data of (**A**) PUM and (**B**) dPUM.


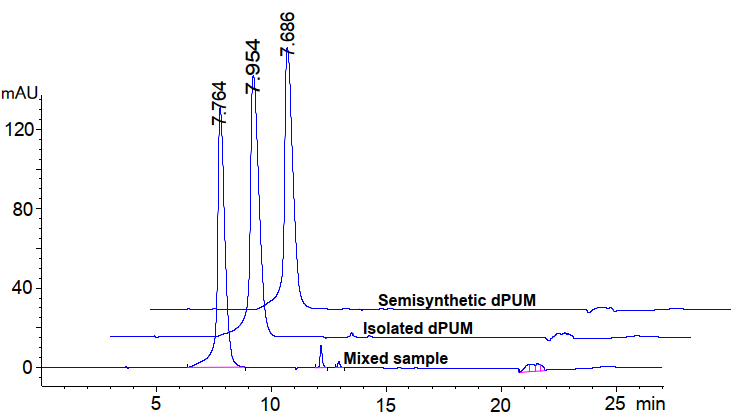


**Figure S16.** HPLC-traces of isolated and semisynthetic desoxy-PUM and a mixed sample containing the compound from both sources.

**Table S3.** ^1^H-NMR data reported for STU and PUM side by side with the data measured in the present study.

| ^1^H-NMR data, DMSO-d_6_ | | | |
| --- | --- | --- | --- |
|  | Rep. STU^a^ (δ, ppm) | Rep. PUM^b^ (δ, ppm) | Measured (δ, ppm) |
| 1´ | 4.37 | 4.34 | 4.42 |
| 2´ | 3.85 | 3.91 | 3.94 |
| 3´ | 3.64 | 3.65 | 3.74 – 3.79 |
| 4´ | 3.77 | 3.65 | 3.74 – 3.79 |
| 5´ 5´´ | 3.16; 3.52 | 3.24;3.14 | 3.30; 3.23 |
| 6´ | 8.44 | 7.82 | 8.01 |
| 1 | / | 10.8 | / |
| 2 |  |  |  |
| 3 | / | 11.04 | / |
| 4 |  |  |  |
| 5 |  |  |  |
| 6 | 7.34 | 7.33 | 7.4 |
| Gln C=O |  |  |  |
| Gln α | 4.77 | 4.72 | 4.73 |
| Gln β | 1.99 | 2.03; 1.90 | 2.13 – 1.87 |
| Gln γ | 2.07 | 2.04; 1.91 | 2.13 – 1.87 |
| Gln δ |  |  |  |
| Gln N*_ε_* | 7.3; 6.77 | 7.26; 6.81 | 7.29; 6.76 |
| Gly α | 4.2 | 4.22;4.12 | 4.23 |
| Gly NH | / | 7.43 | / |
| Gly C=O |  |  |  |
| Guanid. |  |  |  |

/ = not assigned or detected. **a.** Pesic, A. *J. Antibiot.*  **67,** 471–477 (2014). **b.** 1. Maffioli, S. I. *et al*. *Cell* **169**, 1240–1248 (2017).

**Table S4.** ^13^C-NMR data reported for STU and PUM side by side with the data measured in the present study.

| ^13^C-NMR data, DMSO-d_6_ | | | |
| --- | --- | --- | --- |
|  | Rep. STU^a^ (δ, ppm) | Rep. PUM^b^ (δ, ppm) | Measured (δ, ppm) |
| 1´ | 79.9 | 79.8 | 79.6 |
| 2´ | 71.8 | 73.8 | 74 |
| 3´ | 73.8 | 76.3 | 72.6 |
| 4´ | 81.3 | 81.4 | 81.5 |
| 5´ 5´´ | 40.7 | 42 | 41.7 |
| 6´ |  |  |  |
| 1 |  |  |  |
| 2 | 155.9 | 152 | 151.7 |
| 3 |  |  |  |
| 4 | 165.4 | 164 | 164 |
| 5 | 109.1 | 110.7 | 111.1 |
| 6 | 145.4 | 140.4 | 140.4 |
| Gln C=O | 169.8 | 169.5 | 169.9 |
| Gln α | 59 | 59 | 59.7 |
| Gln β | 23.9 | 23.6 | 24.1 |
| Gln γ | 31.8 | 31.7 | 32.3 |
| Gln δ | 174.1 | 174 | 174.5 |
| Gln N*_ε_* |  |  |  |
| Gly α | 42.1 | 43 | 42.8 |
| Gly NH |  |  |  |
| Gly C=O | 168.1 | 157 | 164.4 |
| Guanid. | 157.6 | / | 158.2 |

/ = not assigned or detected. **a.** Pesic, A. *J. Antibiot.*  **67,** 471–477 (2014). **b.** 1. Maffioli, S. I. *et al*. *Cell* **169**, 1240–1248 (2017).
